# Oil accumulation in the model green alga *Chlamydomonas reinhardtii*: characterization, variability between common laboratory strains and relationship with starch reserves

**DOI:** 10.1186/1472-6750-11-7

**Published:** 2011-01-21

**Authors:** Magali Siaut, Stéphan Cuiné, Caroline Cagnon, Boris Fessler, Mai Nguyen, Patrick Carrier, Audrey Beyly, Fred Beisson, Christian Triantaphylidès, Yonghua Li-Beisson, Gilles Peltier

**Affiliations:** 1 Commissariat à l'Energie Atomique et aux Energies Alternatives (CEA), Direction of Life Sciences, Institute for Environmental Biology and Biotechnology, Laboratory of Bioenergetics and Biotechnology of Bacteria and Microalgae, CEA Cadarache, 13108 Saint-Paul-lez-Durance, France; 2 Centre National de la Recherche Scientifique (CNRS), UMR 6191, CEA Cadarache, 13108 Saint-Paul-lez-Durance, France; 3 Aix Marseille Université, Department of Plant Biology and Environmental Microbiology, CEA Cadarache, 13108 Saint-Paul-lez-Durance, France; 4 Current Address: Fermentalg SA, 33500 Libourne, France

## Abstract

**Background:**

When cultivated under stress conditions, many microalgae species accumulate both starch and oil (triacylglycerols). The model green microalga *Chlamydomonas reinhardtii *has recently emerged as a model to test genetic engineering or cultivation strategies aiming at increasing lipid yields for biodiesel production. Blocking starch synthesis has been suggested as a way to boost oil accumulation. Here, we characterize the triacylglycerol (TAG) accumulation process in Chlamydomonas and quantify TAGs in various wild-type and starchless strains.

**Results:**

In response to nitrogen deficiency, *Chlamydomonas reinhardtii *produced TAGs enriched in palmitic, oleic and linoleic acids that accumulated in oil-bodies. Oil synthesis was maximal between 2 and 3 days following nitrogen depletion and reached a plateau around day 5. In the first 48 hours of oil deposition, a ~80% reduction in the major plastidial membrane lipids occurred. Upon nitrogen re-supply, mobilization of TAGs started after starch degradation but was completed within 24 hours. Comparison of oil content in five common laboratory strains (CC124, CC125, *cw15*, CC1690 and 11-32A) revealed a high variability, from 2 μg TAG per million cell in CC124 to 11 μg in 11-32A. Quantification of TAGs on a cell basis in three mutants affected in starch synthesis (*cw15sta1-2, cw15sta6 *and *cw15sta7-1*) showed that blocking starch synthesis did not result in TAG over-accumulation compared to their direct progenitor, the arginine auxotroph strain *330*. Moreover, no significant correlation was found between cellular oil and starch levels among the twenty wild-type, mutants and complemented strains tested. By contrast, cellular oil content was found to increase steeply with salt concentration in the growth medium. At 100 mM NaCl, oil level similar to nitrogen depletion conditions could be reached in CC124 strain.

**Conclusion:**

A reference basis for future genetic studies of oil metabolism in Chlamydomonas is provided. Results highlight the importance of using direct progenitors as control strains when assessing the effect of mutations on oil content. They also suggest the existence in Chlamydomonas of complex interplays between oil synthesis, genetic background and stress conditions. Optimization of such interactions is an alternative to targeted metabolic engineering strategies in the search for high oil yields.

## Background

Bioethanol and biodiesel are currently produced from food crops such as sugar beet, sugar cane, soybean or rapeseed, thus competing with land use for food production and urging to find new feedstocks [1]. Due to their high surface productivity, microalgae are considered as a promising way of producing biofuels [2-4]. Under standard growth conditions, microalgal biomass is mainly composed of proteins, cell wall carbohydrates and membrane lipids. Accumulation of energy-rich reserve compounds such as starch and storage lipids (oil) occurs in many microalgae under conditions of nutrient shortage such as nitrogen (N) deficiency [3,5].

Oil is largely composed of long-chain triacylglycerols (TAG) and represents a form of energy storage 2.25 times greater than starch on a weight basis [6]. TAGs can be converted to biodiesel by chemical transesterification of its fatty acids and is thus a highly desirable storage compound. Some microalgae species accumulate up to 50% TAG on a dry weight basis in response to N deficiency [2,7]. Despite high biomass productivity and ability to accumulate high oil amounts, microalgal biodiesel is currently not competitive for several reasons. Firstly, contamination of microalgal cutltures by bacteria, viruses and other microalgae is a common issue. Secondly, the cost of microalgae cultivation, biomass harvest and oil extraction contribute significantly to the overall costs. Thirdly, intracellular oil accumulation requires a phase of nutrient starvation, which severely decreases the overall productivity of the system [2,3]. It is therefore clear that competitiveness of microalgal biodiesel will depend on improvements in both cultivation and harvesting technologies [4] as well as in strain performances [3].

Improving microalgal strain performances requires a better understanding in model microalgae of the mechanisms and regulations of carbon fixation, carbon allocation between biosynthetic pathways and induction by stresses. The green unicellular alga *Chlamydomonas reinhardtii *is a widely recognized model organism to investigate numerous biological functions, including photosynthesis [8], starch metabolism [5,9] or flagella [10]. The recent sequencing of its whole genome [11], the availability of numerous molecular tools including transformation of the three (nuclear, plastid and mitochondrial) genomes, and the existence of a sexual cycle allowing genetic studies make *C. reinhardtii *an attractive model for molecular investigations [12]. In addition to its well known starch reserves, *C. reinhardtii *has also been observed to accumulate intracellular oil droplets under N limiting conditions [13,14]. Pathways of TAG biosynthesis are still poorly documented in microalgae including in *C. reinhardtii *and most putative reactions are based on similarity of microalgal sequences to characterized proteins from bacteria, yeasts and higher plants [15]. Starch biosynthesis on the other hand has been particularly well-studied in this organism due to the isolation of several starchless mutants [13].

Whether starch synthesis competes with oil synthesis for carbon precursors is an important question. If such a competition exists, shutting down starch biosynthesis could be a simple way to increase the amount of oil stored in microalgal cells. Increased amount of oil on a dry weight basis has been reported for starchless mutants in *Chorella pyrenoidosa *[16] and more recently in *Chlamydomonas reinhardtii *[17] but no direct quantitative estimates of the oil content per cell were provided. Another study reports a 1.5 to 2.0 fold increase in oil per cell in a Chlamydomonas starchless mutant [18], which suggests a competition between oil and starch syntheses. However, recent data showing that complemented strains of *C. reinhardtii *starchless mutants have both high oil and high starch content [19] seems inconsistent with a competition hypothesis. The first studies of oil mutants in Chlamydomonas thus highlight the gaps existing in our understanding of oil deposition in this species and on the factors that might be critical when assessing the effect of mutations on oil content. Gaining insights into these issues is important because Chlamydomonas will increasingly be used as a model to study oil synthesis and isolate oil mutants.

Here, we characterize the oil accumulation process in *C. reinhardtii *by investigating the kinetics of oil deposition and mobilization comparatively to starch, as well as the changes occurring in major plastidial membrane lipids during TAG accumulation. We also show that common laboratory strains of Chlamydomonas widely used as references in mutant comparisons have up to 5-fold variation in their capacity to accumulate oil. Comparison of starchless mutants using appropriate reference strain on a per cell basis shows that blocking starch synthesis has no significant effect on oil accumulation in the *cw15 *background. Finally, it is shown that in *C. reinhardtii *CC124 (137C) wild-type strain, oil accumulation can be induced by salt stress, which could advantageously replace nitrogen depletion in mutant screens.

## Results

### Cellular oil content varies up to 5-fold in common laboratory strains of *C. reinhardtii*

**Figure 1:**
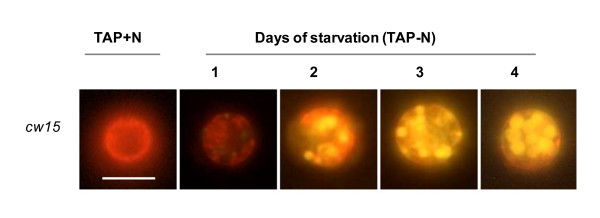
**Detection of neutral lipid accumulation in *cw15*, a common laboratory strain of *C. reinhardtii *using Nile red staining**. The yellow fluorescence observed in the presence of Nile red indicates the presence of neutral lipids while the red fluorescence corresponds to chlorophyll autofluorescence. TAP: standard growth medium. TAP-N: nitrogen-depleted TAP medium. Bars = 8 μm.

Like many microalgae, *C. reinhardtii *accumulates starch when cultivated in N-depleted medium [9]. Recently, it has become clear that under these stress conditions *C. reinhardtii *also synthesize neutral lipids [14,18]. Accumulation of these compounds can be monitored by a simple stain with Nile red, a fluorescent dye emitting a yellow fluorescence signal (around 580 nm) in the presence of neutral lipids [20]. Using this simple technique, accumulation of neutral lipids was followed during a 4-day N starvation in *cw15*, a common laboratory strain of *C. reinhardtii*. Clear qualitative differences in the level of neutral lipids accumulated within cells could be seen before and after nitrogen removal (Figure 1).

**Figure 2:**
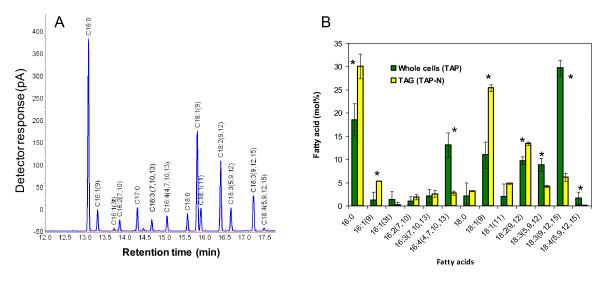
**Analysis of fatty acid composition in *C. reinhardtii***. (A) Separation by GC-FID of fatty acid methyl esters prepared from total lipids of *C. reinhardtii cw15*. (B) Comparison of the fatty acid composition of the TAG fraction and total lipids. The TAG fraction was isolated from cells cultivated in TAP-N for 2 days while total lipids were from cells grown in TAP medium. Values are mean of four independent experiments ± SD. Asterisks denote a statistically significant difference (t test, two-sided P < 0.05 at least).

To determine if the neutral lipids detected were indeed triacylglycerols (TAGs), total cellular lipids were extracted and separated by high performance thin layer chromatography (HPTLC). The TAG fraction was identified by co-migration with a purified TAG standard, recovered from the HPTLC plate and its fatty acid content analyzed by gas chromatography with flame ionization detection (GC-FID) (Figure 2A). *C. reinhardtii *TAGs induced by N depletion had a fatty acid composition dominated by 16:0 (palmitic acid), 9c-18:1 (oleic acid) and 9c,12c-18:2 (linoleic acid), which is similar to many plant oils. This was in clear contrast with the fatty acid profile of *C. reinhardtii *whole cells grown under standard conditions (TAP medium), whose major lipid species were membrane lipids and were thus rich in polyunsaturated species such as 9c,12c,15c-18:3 (α-linolenic acid), 4c,7c,10c,13c-16:4, 5c,9c,12c-18:3, and 5c,9c,12c,15c-18:4 (Figure 2B)

**Figure 3:**
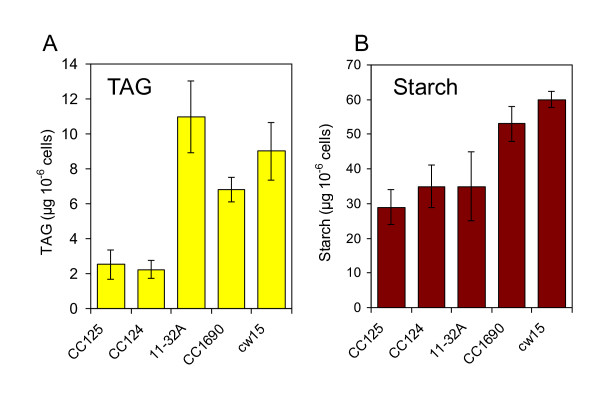
**Triacylglycerol and starch accumulation in common laboratory strains of *C. reinhardtii***. (A) Triacylglycerols, (B) Starch. Cells were analyzed after culture in TAP-N for 2 days; values are mean ± SD, n = 4.

Amount of TAGs measured by HPTLC/densitometry and HPTLC/GC-FID were not significantly different (data not shown). Densitometry was therefore used routinely to quantify TAGs. Oil content was measured for *cw15*, CC124 as well as three other strains grown at the same time in flasks agitated in an incubator where light intensity and temperature were controlled. While under standard growth conditions (TAP medium) all strains were found to store low TAG amounts (<1 μg 10^-6 ^cells), all strains accumulated TAGs in response to a 2-day nitrogen depletion, but interestingly not to the same levels (Figure 3A). TAG amounts varied over 5-fold, ranging from about 2 to 11 μg per million cell in CC124 and11-32A respectively, with intermediate levels of TAGs found in other strains. These five laboratory strains also showed a variability in their capacity to accumulate starch following nitrogen deprivation, but the variation in starch content was clearly lower than for oil (2-fold, from 30 to about 60 μg per million cell) (Figure 3B).

### The bulk of plastidial membrane lipids is degraded at the beginning of the oil accumulation phase

**Figure 4:**
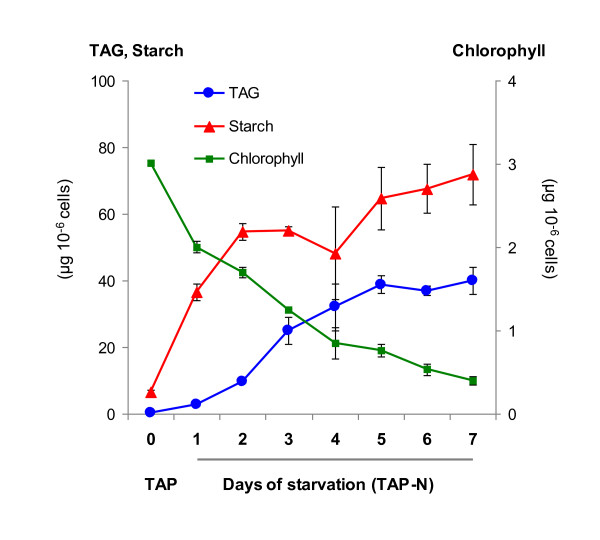
**Time course of accumulation of triacylglycerols, starch and chlorophyll in *C. reinhardtii *in response to nitrogen depletion**. Precultures of strain *cw15 *were grown in TAP medium for 2 days before changing medium to TAP-N (day 0). Values are mean of three independent experiments ± SD.

To characterize better the reserve deposition process triggered by nitrogen deprivation, a strain with high TAG content was chosen (*cw15*) for further studies. Amounts of starch, oil and chlorophyll per cell were followed for 7 days after N removal. While starch accumulation was observed after 1 day following nitrogen depletion (TAP-N media) and had already reached high levels by day 2 (about 60 μg per million cell), TAGs started to accumulate at a much slower rate, reaching maximal level after 5 days only (40 μg per million cell) (Figure 4). Phenotypically, nitrogen-starved cells had a yellowish appearance compared to those grown under nutrient replete conditions. Quantification of chlorophyll content showed a sharp decrease from 3 μg 10^-6 ^cells to 0.5 μg 10^-6 ^cells as N deficiency progressed (Figure 4). A similar pattern of lipid accumulation was also observed in the low-oil accumulator CC124 (see additional file 1).

**Figure 5:**
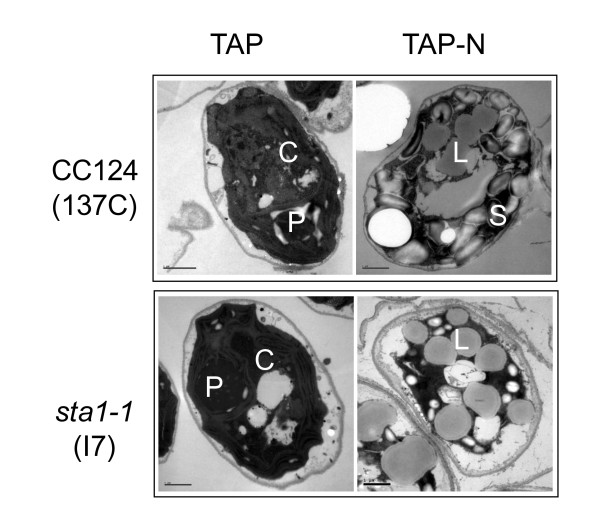
**Transmission electron microscopy images of *C. reinhardtii *CC124 and starchless mutant *sta1-1 *before and after 3 days of nitrogen depletion**. Bars = 1 μm. P: pyrenoid; C: chloroplast; L: lipid droplet, S: starch granule.

Changes in cellular structures caused by nitrogen depletion were observed by transmission electron microscopy. In *C. reinhardtii*, a single cup-shaped plastid takes up over two thirds of the total cellular volume when cultivated under nutrient replete conditions [21], where neither starch granule nor lipid bodies could be detected. The accumulation of starch granules and oil-bodies as well as a reduction in plastidial membranes were evident in N-starved wild-type cells (Figure 5). Taken together, these observations suggest that N depletion causes a reduction in the photosynthetic apparatus and a breakdown of plastidial membranes.

**Figure 6:**
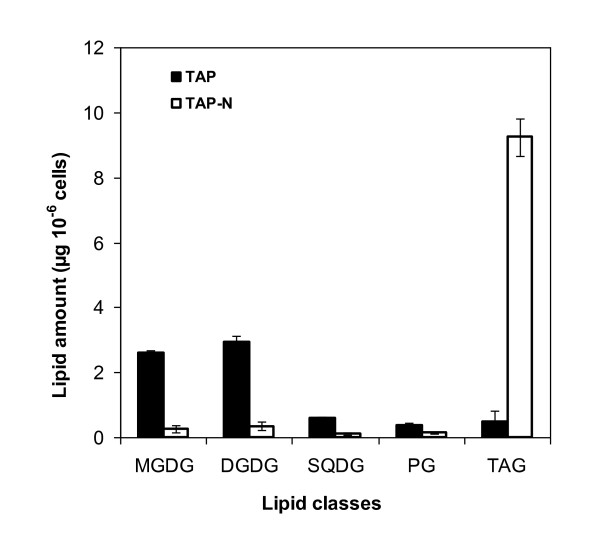
**Changes in triacylglycerols and major plastidial lipid classes in nitrogen-starved cells**. Cells were cultivated in TAP-N for 2 days. Values are mean of four independent experiments ± SD.

Separation and quantification of the major membrane lipids showed that the bulk of plastidial lipids were indeed reduced drastically (Figure 6). At day 2, while TAG amount had increased over 15-fold, there was a >80% reduction for monogalactosyl diacylglycerol (MGDG), digalactosyl diacylglycerol (DGDG) and sulfolipid sulfoquinovosyl-diacylglycerol (SQDG). Consistently, a major change in the fatty acid profile of total lipids (a decrease in polyunsaturated species and an increase in saturated and monounsaturated ones) was observed within the first 24 hours following N depletion in the culture medium (additional file 2). These results indicate that membrane breakdown does not take place at a constant rate during the 5 days of the TAG accumulation process but occurs massively at the beginning.

### No over-accumulation of TAGs occurs in *cw15*-derived starchless mutants compared to their progenitor

**Figure 7:**
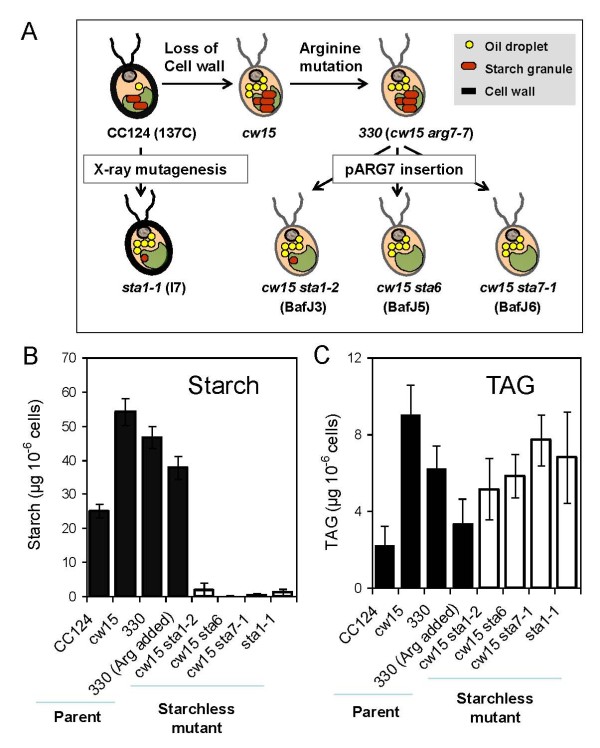
**Comparison of oil and starch reserves in various strains of *C. reinhardtii***. (A) Relationships between the wild-type and starchless strains tested. Presence of triacylglycerol and starch reserves in N-starved cells is illustrated. Direct progenitors of the mutants are indicated. Loss of the cell wall in some strains is also highlighted. (B, C) Quantification of starch and triacylglycerol accumulation in starchless mutants (*sta1-1, sta1-2, sta6*, and *sta7-1*) and its progenitors (CC124, *cw15*, *330*). Culture conditions: TAP-N for 2 days. Values are mean of four independent experiments ± SD.

Under N depletion conditions, starch represents up to 40% and 80% dry weight in the *C. reinhardtii *strains CC124 (137C) and *cw15 *respectively. Based on oil content per dry weight, it has been suggested that shutting down starch synthesis results in higher oil accumulation capacity [17,22]. Given the high variability in TAG level observed in various common laboratory strains (Figure 3) several starch mutants were checked and direct progenitors were used as reference strains for assessing the effect of shutting down starch biosynthesis on oil content. Mutant strains included *sta1-1 *(I7), *sta1-2 *(BafJ3), *sta6 *(BafJ5) and *sta7-1 *(BafJ6) (Figure 7A). All these mutants, which were obtained from the Ball laboratory (University of Lille, France), either do not make starch at all, or make less than 5% of the wild-type level [13,23-25]. The expected absence or reduced amount of starch in the mutants was confirmed (Figure 7B). For the sake of simplicity, in this paper we refer to all these strains as 'starchless'. Except *sta1-1*, which is derived from wild-type strain CC124 (137C) by X-ray mutagenesis and has a cell wall, the other mutants were obtained from the wall-less arginine auxotroph *330 *(*cw15 arg7-7*) by insertional mutagenesis and do not have a cell wall. Therefore, the three insertional mutants are affected in both starch and cell wall and are named *cw15sta1-2*, *cw15sta6 *and *cw15sta7-1 *in this study. Detailed genotypes and references of all strains used are outlined in Table 1.

**Table 1:** Genotypes and references of *Chlamydomonas reinhardtii *strains used

Classes	Strains	Other names	Genotype	References or Source
Wild type	CC124	137C *mt-*	*mt- nit1 nit2*	Chlamydomonas Center

Wild type	CC125	137C *mt+*	*mt+ nit1 nit2*	Chlamydomonas Center

Wild type	11-32A		*mt+*	CCAP collection

Wild type	CC1690		*mt+*	Chlamydomonas Center

Wild type	*cw15 mt-*	CC406	*mt- cw-*	Chlamydomonas Center

Wild type	*cw15 mt+*	CC400	*mt+ cw-*	Chlamydomonas Center

Wild type	*330*		*mt+ nit1 nit2 cw15 arg7-7*	Lab of Steven Ball

Starchless Mutants	*sta1-2*	BafJ3	*mt+ nit1 nit2 cw15 arg7-7 sta1-2::ARG7*	[24]

Starchless Mutants	*sta7-1*	BafJ6	*mt+ nit1 nit2 cw15 arg7-7 sta7-1::ARG7*	[23]

Starchless Mutants	*sta1-1*	I7	*mt- nit1 nit2 sta1-1*	[13]

Starchless Mutants	*sta6*	BafJ5	*mt+ nit1 nit2 cw15 arg7-7 sta6-1::ARG7*	[25]

BafJ5 complemented lines	BafJ5C2		*mt+ nit1 nit2 cw15 arg7-7 sta6-1::ARG7 pSL18-STA6*	Lab of Steven Ball

BafJ5 complemented lines	BafJ5C3		*mt+ nit1 nit2 cw15 arg7-7 sta6-1::ARG7 pSL18-STA6*	Lab of Steven Ball

BafJ5 complemented lines	BafJ5C6		*mt+ nit1 nit2 cw15 arg7-7 sta6-1::ARG7 pSL18-STA6*	Lab of Steven Ball

BafJ5 complemented lines	BafJ5C7		*mt+ nit1 nit2 cw15 arg7-7 sta6-1::ARG7 pSL18-STA6*	Lab of Steven Ball

BafJ5 complemented lines	BafJ5C8		*mt+ nit1 nit2 cw15 arg7-7 sta6-1::ARG7 pSL18-STA6*	Lab of Steven Ball

BafJ5 complemented lines	BafJ5C9		*mt+ nit1 nit2 cw15 arg7-7 sta6-1::ARG7 pSL18-STA6*	Lab of Steven Ball

BafJ5 complemented lines	BafJ5C16		*mt+ nit1 nit2 cw15 arg7-7 sta6-1::ARG7 pSL18-STA6*	Lab of Steven Ball

BafJ5 complemented lines	BafJ5C18		*mt+ nit1 nit2 cw15 arg7-7 sta6-1::ARG7 pSL18-STA6*	Lab of Steven Ball

BafJ5 complemented lines	BafJ5C20		*mt+ nit1 nit2 cw15 arg7-7 sta6-1::ARG7 pSL18-STA6*	Lab of Steven Ball

**Figure 8:**
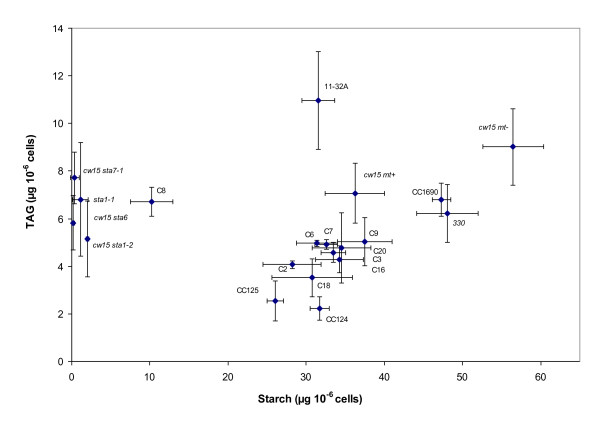
**Absence of correlation between oil and starch content in various *Chlamydomonas reinhardtii *strains**. C2,3,6,7,8,9,16,18,20 are independent lines of complementants for *sta6 *(BafJ5). Mean contents in starch and oil were calculated based on at least three independent experiments. Error bars indicate standard deviations. Mean content in oil and starch were subjected to a Kendall rank correlation test. The null hypothesis that oil and starch are independent cannot be rejected (tau statistics = 0.07; two-tailed p = 0.673; n = 20).

Quantification of TAGs after 2 days of nitrogen depletion in the four starchless mutant strains showed that the oil content per cell was significantly higher than their distant parent, the low oil accumulator CC124 (Figure 7C). However, in the three mutants that are in the *cw15 *background, the oil content was clearly not higher than in *cw15 *or in their direct progenitor *330*. When oil content was measured 4 days after N depletion, there was still no difference between the starchless mutants and their progenitor *330 *(data not shown). This clearly showed that in the *cw15 *background blocking starch synthesis did not result in over-accumulation of oil compared to the progenitor strain. TAG content was further analyzed in 9 independent complemented lines of the *cw15sta6 *mutant. Eight of these lines showed almost complete complementation for starch content (>30 μg per million cell compared to about 50 μg in the wild-type progenitor *330*). Almost all these eight lines had an oil content per cell not significantly lower than that of the *cw15 sta6 *mutant (Figure 8). This confirmed that the *sta6 *mutation did not cause TAG over-accumulation. Moreover, when all the wild-type mutant and complemented strains tested were compared, no significant correlation between oil and starch content was found (Figure 8). Most importantly, the C8 line partially complemented for starch (10 μg per million cell vs. 50 μg in *330*) did not show higher oil than *cw15 *or *330*. Taken together these results argued strongly against a competition between oil and starch synthesis in the *cw15 *background.

It should be noted that in these experiments arginine was added to the preculture medium of the control strain *330 *(this strain is auxotroph for arginine). However, arginine was omitted from the N-depleted medium. Indeed, arginine is a source of nitrogen for *C. reinhardtii *[26,27] and would create a bias in the comparison of *330 *to the starchless mutants. As shown on Figure 7B, the addition of arginine in TAP-N cultures at the concentration normally used for precultures (100 μg mL^-1^) reduced the content of oil in *330 *by half compared to the TAP-N medium without arginine added. This reduction in oil content in response to arginine addition was also observed in *cw15 *strain (data not shown).

### Oil and starch accumulated during N starvation phase are rapidly mobilized upon switching to nutrient replete conditions

**Figure 9:**
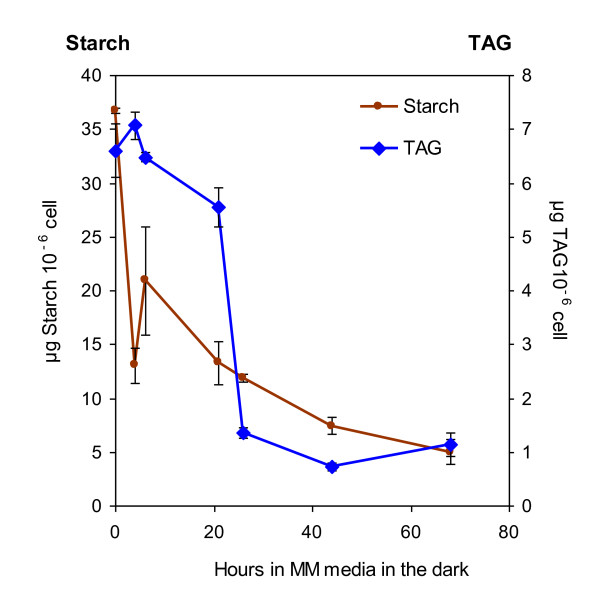
**Mobilization of triacylglycerols and starch in strain *330 *after re-supply with nitrogen**. Cells were first cultivated in TAP medium until mid log phase, then transferred to TAP-N for 3 days under constant light and then switched to MM media in the dark. Values are mean of three independent experiments ± SD.

To gain more insights into the dynamics of oil and starch reserves and find conditions that will be useful to screen for mutants impaired in oil degradation, experimental conditions causing TAG breakdown were sought and kinetics of mobilization were determined. *C. reinhardtii *cells were first starved for 3 days under TAP-N in the light to induce accumulation of reserves. Then, to create a need for carbon source and achieve conditions likely to involve reserve mobilization, cells were transferred to Tris-Minimal Media (MM) with nitrogen, and kept in the dark for 3 days. Cellular oil and starch content was followed during this dark period. Starch degradation was found to occur very rapidly after switching to dark and started earlier than oil degradation (Figure 9). After 20 hours, 70% starch had already been catabolised to support growth while oil breakdown only begun. The bulk of oil was degraded between 20 and 24 hours of N resupply. Starch degradation continued between 20 and 60 hours while TAGs remained constant at ~1 μg 10^-6 ^cells, a level equivalent to that of healthy grown un-starved cells. After 24 hours cells became green again (data not shown). The apparent decrease in starch and oil content per cell was not due to dilution by cell division because expression on a per mL culture resulted in similar results (additional file 3). It thus appears that *C. reinhardtii *can use both oil and starch but that there is a differential mobilization of these two types of reserves. The observation that starch is accumulated as well as degraded at a faster rate than the accumulation/degradation of TAGs is consistent with the view that in Chlamydomonas starch and oil have different purposes, starch being the reserve preferentially synthesized and mobilized and oil representing a long term storage in case of prolonged shortage or stress.

### Salt stress can induce oil accumulation in *C. reinhardtii *CC124

So far, the most widely used stress to trigger oil accumulation in microalgae is the removal of nitrogen from the culture medium. Other stress conditions such as high pH, salinity, light or extreme temperatures have also been described as triggering TAG accumulation, but quantitative data are still lacking for *C. reinhardtii *[3].

**Figure 10:**
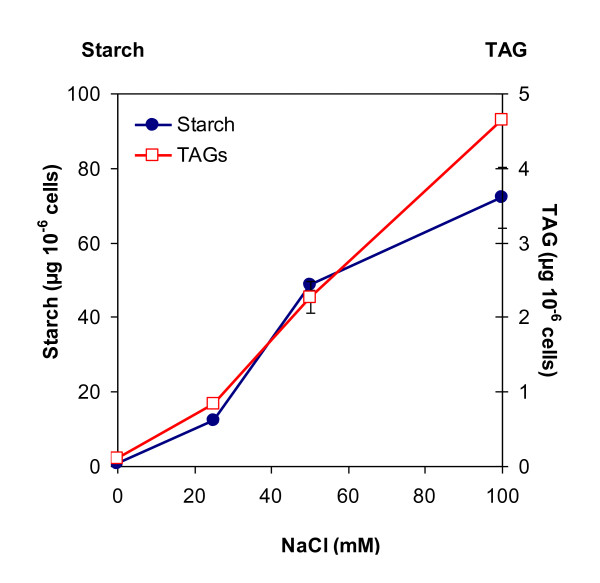
**Effect of salt supplementation on starch and triacylglycerol content in *C. reinhardtii***. Wild-type strain CC124 was used. Cells were first cultivated in standard TAP medium reaching mid-log phase. Different NaCl amounts were then added to the cultures, which were further grown for 2 days before samples were taken for starch and lipid analyses. Values are mean of three independent experiments ± SD.

High salt (at 1 M) has been reported to increase intracellular accumulation of TAGs by about 65% in cells of the marine microalga *Dunaliella salina *[28]. In order to test the effect of salt stress on the accumulation of storage compounds in *C. reinhardtii*, TAP culture media containing nitrogen were supplemented for 2 days with 0, 20, 50, and 100 mM NaCl. Cell wall-less strain *cw15 *was not a strain of choice for this experiment because the absence of cell wall makes it more sensitive to high osmotic pressure. Thus, wild-type CC124 was used. We observed that both starch and TAG reserves increase with NaCl concentrations in TAP cultures (Figure 10). At 100 mM NaCl, starch content was reached around 70 μg per million cell, which was 4-fold higher than in TAP-N medium. TAGs went up to about 5 μg per million cell, a level similar to that reached by this strain under N deprivation. Under these salt stress conditions, growth was stopped as observed for N deprivation.

## Discussion

How to trigger oil synthesis and increase oil content in vegetative cells (plant leaves or microalgae) is a hot topic in many industrial and academic labs across the globe. In this study, we characterize oil accumulation in the model green microalgae *Chlamydomonas reinhardtii *and show that the level of oil accumulation caused by nitrogen deficiency in common laboratory strains is highly dependent on the strain used. The comparison of the oil content in various starchless mutants and their direct progenitors in two different genetic backgrounds reveals that carbon allocation between starch and oil pathways in N-starved Chlamydomonas cells is likely to be more complex than previously thought. Substitution of N depletion by salt stress highlights the importance of culture conditions and stress perception in building oil reserves.

### Factors critical for assessing effect of mutations on oil content

**Figure 11:**
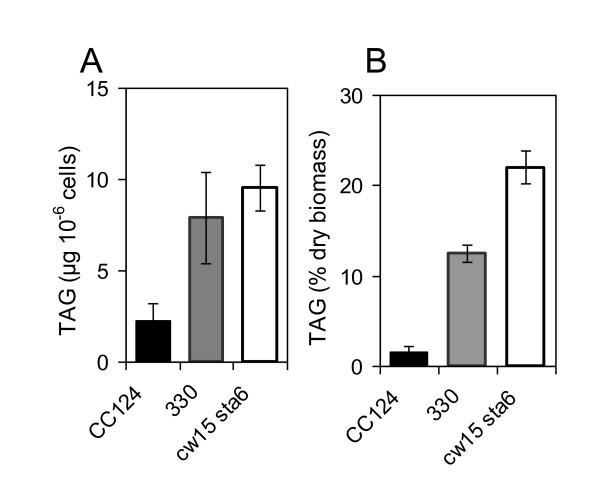
**Influence of reference basis and control strains when comparing oil content**. TAG amount is expressed on a μg 10^-6 ^cells basis (A) or a % dry weight basis (B). Two reference strains, CC124 (137C) and the cell wall-less *330 *(*cw15arg7-7*) are shown. Cells were cultured in TAP-N for 2 days. Values are mean of four independent experiments ± SD.

Several reports on Chlamydomonas mutants affected in oil content have been recently published. Li et al [17,22] showed that on a dry weight basis the oil content in the starchless and cell wall-less mutant *cw15sta6 *(BafJ5) is 10-fold higher than the wild-type strain CC1690 containing a cell wall. Work et al [19] observed that *cw15sta6 *makes 2-fold more oil on a cell basis compared to the cell wall-containing and low oil accumulator CC124 (137C) strain. Wang et al [18] reported that after 18 hour of N-depletion, *cw15sta6 *make 1.5-2.0 times more oil on a cell basis than an arginine revertant or a suppressed clone of *330*. Data on starchless mutants presented here highlight that two factors are critical when comparing oil contents: *i) *the strain used as a control (*i.e. *direct progenitor or other strain) and *ii) *the basis on which data are expressed (i.e. per cell or per dry weight). Conclusion drawn from oil content comparisons can be different and even opposite depending on the reference basis and control strain used. This is illustrated on Figure 11. Concerning the control strain, it is clear that if one uses a reference strain with cell wall such as CC124 (137C) (Figure 11A), the conclusion drawn will be that *sta6 *mutation is responsible for increased oil content in *cw15sta6 *as recently suggested [17,19]. However, comparison using the direct progenitor *330 *(*cw15arg7-7*) shows that there is no significant effect of the *sta6 *mutation on oil accumulation (Figure 11A).

The importance of reference basis is also illustrated on Figure 11: the mutant strain *cw15 sta6 *makes about twice more oil than *330 *on a dry weight basis (Figure 11B) but on a cell basis it makes similar amount of oil (Figure 11A). This is actually not surprising because starch makes significant contribution to cell dry weight (over 50% dry weight in *330*). Although informative from the point of view of the enrichment of the biomass in oil, the dry weight basis can be misleading when assessing the effect of engineering strategies on the metabolism of a cell. Indeed, mutant strains might show large differences in dry weight per cell compared to wild-type. The per cell basis is thus more appropriate for metabolic engineering comparisons because it reflects the actual capacity of the oil biosynthesis pathway in cells, independently of other products such as cell wall and starch which might contribute very substantially to dry weight.

### Interaction between starch and oil depositions

Starch and oil are two major carbon sinks in growth arrested cells [29]. The idea of a competition between starch and oil syntheses is clearly not supported by the fact that in the cell wall-less background *cw15 *no increase in oil was observed in three starchless mutants compared to their direct progenitor or to complementants. In the cell wall containing CC124 (137C) background, the starchless mutant *sta1-1 *has about 3-fold more oil than its progenitor CC124 (Figure 7C). It should be noted however that there is no evidence that the increase in oil is caused by the *sta1-1 *mutation. *Sta1-1 *has been obtained by X-ray mutagenesis and might carry many additional mutations affecting oil content. The high variability in oil observed in wild-type strains suggests that mutations affecting oil content are common. Unfortunately, genetic analysis cannot be performed to prove that *sta1-1 *mutation is responsible for increased oil because *sta1-1 *mutant cannot be crossed. In summary, although it cannot be completely ruled out that the effect of blocking starch synthesis is dependent on the genetic background, current evidence on mutants and complementants in *cw15 *background shows that blocking starch synthesis does not result in over-accumulation of oil in Chlamydomonas cells. Therefore, allocation of C precursors to reserves in N-starved *C. reinhardtii *seems to involve more subtle relationships than a mere competition between oil and starch syntheses. This view is also clearly supported by the fact that the high oil accumulator 11-32A has normal level of starch (Figure 3). Another evidence of complexity of carbon partitioning comes from the observation that complemented mutant lines of *cw15sta7-10 *not only restored their wild-type starch levels, but also make more oil than the mutant [19].

The idea that impairement of starch synthesis in photosynthetic organisms does not necessarily result in higher oil content is also illustrated by data in higher plants. In *Pisum sativum*, a starchless mutant (*rug3*) defective in the plastid phosphoglucomutase gene which has almost lost all its starch reserve, was found to have a significantly increased seed lipid content [30], whereas mutation in the Arabidopsis ortholog (*pgm-1*) leads to a 40% reduction in oil content compared to wild-type seeds [31]. A transient starch accumulation has been postulated to provide a carbon source for lipid synthesis during accumulation phase in plant photosynthetic tissues [32]. This hypothesis is yet to be tested in *C. reinhardtii*.

### Origin of the high variability in oil content in common laboratory strains of Chlamydomonas

High variation in oil content in Chlamydomonas reference strains is clearly an issue that has been overlooked so far and, as discussed above, has important practical applications when comparing oil contents in mutants. Concerning the cause of this variation between laboratory strains (which all originate from the same 1945 isolate of G.M. Smith) [33], we can only speculate that among the mutations accumulating in the haploid cells of Chlamydomonas during growth and storage in laboratories, many factors affect oil indirectly (possibly because TAG synthesis is a response to stress and adverse conditions). *C. reinhardtii *is a soil dwelling alga [21]. As for other unicellular or pluricellular organisms, accumulation of TAGs might have important roles for cell survival in some Chlamydomonas strains in their natural environments. In their natural habitat, microalgae often accumulate oil and/or starch in adverse circumstances such as a nutrient shortage. When the conditions become favourable, stored carbon reserves are usually fuelled up to support cell division and vegetative growth. Other putative roles of TAGs in microalgae include energy storage for germination of zygote and flotation/swimming of vegetative cells to the surface of ponds or other habitats, and membrane turnover and detoxification of endogenous or exogenous free fatty acids. Low selective pressure on high TAG content that is applied in strains cultivated under standard growth conditions in the laboratory might have resulted in accumulation of mutations lowering oil content.

Whether the high TAG accumulation observed in the cell-wall deficient strains *cw15 *and *330 *is actually caused by the absence of cell wall is unclear. Possible mechanisms linking cell wall and oil synthesis include increased sensitivity to stress caused by cell wall deficiency, changes in osmolarity or cell turgor, or changes in the nutrient (N) perception and sensing machinery or enzymes located inside the cell wall or in the periplasm [34]. Nonetheless the disturbance in cell wall synthesis triggering oil accumulation is not new and has been previously observed in many diatoms, for example, the deficiency of silica, a major component of diatom cell walls, leads to much more pronounced oil accumulation than any other nutrient deficiency (including nitrogen or phosphate) [35]. Further work on other cell wall-less mutants is clearly needed to prove or disprove a causal relationship between cell wall integrity and oil accumulation.

### Chlamydomonas as a model to study lipid metabolism

Chlamydomonas was originally classified as a microalga not suitable to study oil accumulation based on the low oil content of the strain investigated [36]. Our data show that strains of Chlamydomonas such as *cw15 *and 11-32A accumulate high amounts of oil and are thus good models for further studies aiming at isolating low oil mutants. Even 137C (CC124) could be used for this purpose because the low amounts of TAGs produced by this strain can be measured by the HPTLC we used.

Concerning the isolation of Chlamydomonas lipid mutants, the effect of salt addition provides a convenient alternative to N-depletion procedures for inducing oil accumulation in mutant screens, which are often performed in microplates. Removal of N by progressive depletion cannot be controlled in microplates, and centrifugations to change the medium are difficult or impossible to achieve depending on the strain with or without flagella. The rapid decrease in stored TAGs we have evidenced under certain conditions (Figure 9) could also serve as a basis for a screen aiming at isolating mutants affected in oil degradation. Isolating such mutants is important because continuous TAG degradation could occur and limit oil build up in Chlamydomonas as was shown in plant leaves [37].

Polyunsaturated fatty acids are easily oxidized and thus not desirable for biodiesel. The low content in these fatty acid species in Chlamydomonas shows some microalgal oil could be used for biodiesel without much additional genetic modifications. Further genetic engineering strategies aiming at fatty acyl chain modification could be directed toward expressing short or medium chain fatty acyl-ACP thioesterases to produce other fatty acids such as C10:0-C14:0.

## Conclusions

Crops used today have been selected throughout ages for increased abilities to store reserve compounds such as oil or starch as well as for increased adaptation to agricultural practices [38]. Mass cultivation of microalgae is still in its infancy and will certainly need a domestication of species to improve lipid productivity, but also to adapt strain characteristics to technologies used for mass cultivation. Our data suggest that oil mutants should be assessed very cautiously and that approaches aiming at modifying growth and stress conditions in different genetic backgrounds might constitute interesting alternatives to metabolic engineering strategies in view of increasing oil yields.

## Methods

### Strains and cultivation conditions

*Chlamydomonas reinhardtii *wild-type, low starch and starchless mutant strains and their genotypes used in this study are described in Table 1. Briefly, *sta1-2 *(BafJ3), *sta6 *(BafJ5), and *sta7-1 *(BafJ6) strains were generated from cell wall deficient arginine-requiring strain *330 *(*mt+ nit1 nit2 cw15 arg7-7*) by random integration of cassette pARG7 in the nuclear genome [24,25,39]. Mutant strain *sta1-1 *(I7) is derived by X-ray mutagenesis of wild-type strain CC124 (137C) [13]. Unless stated otherwise, strain *cw15 *used in this study is the mating type *mt-. *Complemented lines of *sta6 *(BafJ5) mutant (BafJ5C2, 3, 6, 7, 8, 9, 16, 18, 20) were generated by complementation with plasmid pSL18-STA6 and transformants selected based on paromomycine resistance. These lines were kindly provided by the Steven Ball lab (University of Lille, France).

Unless stated otherwise, all strains were grown at 24°C in continuous light (150 μmoles photons m^-2 ^s^-1^) in the presence of acetate in liquid cultures under shaking (120 rpm). Standard Tris-Acetate-Phosphate (TAP) medium, which includes 7.5 mM NH_4_Cl, is detailed in [33]. For nitrogen starvation studies, exponential phase (5 × 10^6 ^cells mL^-1^) cultures were centrifuged at 600 g for 5 min at room temperature, cell pellets kept and washed twice in TAP medium without nitrogen (TAP-N). Pellets were then resuspended in TAP-N medium and cells grown under constant light under shaking. For the cell wall-deficient arginine-requiring strain *330*, arginine 100 μg mL^-1 ^was added to the TAP medium but was omitted from TAP-N. To prepare the Tris-Minimal Medium (MM), solution was titrated to pH 7.0 with HCl instead of acetic acid. Cell growth and cell size were monitored by cell counts with automated cell counter (Multisizer™ 3 Coulter Counter, Beckman Coulter, USA).

### Fluorescent microscopy

Nile red is a general fluorescent lipophilic stain [20] widely used to reveal the presence of lipid droplets in various microorganisms or plant parts [40]. *C. reinhardtii *cells were stained by a 20 min incubation in the dark at room temperature in a 0.1 μg mL^-1 ^solution of Nile red (Sigma, Saint Louis, USA). Nile red stock solution was 0.1 mg mL^-1 ^in methanol. In the presence of neutral lipids, Nile red emits a yellow-gold fluorescence (λ_max _= 580 nm) while in the presence of polar lipids Nile red emits an orange-red fluorescence (λ_max _= 615 nm) [20]. Nile red fluorescence was observed with a Leica DMRXA epifluorescent microscope equipped with a 63x/1.6 oil immersion objective (Leica Microsystems, Germany), using ByPass excitation (475/40) and LongPass barrier (510) filters. Images were captured with the Spot Insight 4 software (Diagnostic Instruments Inc., Sterling Heights, USA).

### Transmission electron microscopy

*C. reinhardtii *cells grown in the presence of N were harvested by centrifugation (600 *g*, 10 min) at mid-log phase and, after three days of N starvation, fixed with 2.5% glutaraldehyde in 0.2 M cacodylate buffer (pH 7.4) for 2 days at 4°C and rinsed three times 5 min with 0.2 M cacodylate buffer. Cells were then post-fixed for 1 h at 4°C using 1% osmium tetroxide in 0.2 M cacodylate buffer (pH 7.4), dehydrated in increasing concentrations of ethanol (5 min 70% ethanol, 10 min 95% ethanol and 10 min 100% ethanol) and embedded in EMBED 812. Ultrathin sections of either 70 or 90 nm were stained with uranyl acetate followed by lead citrate and examined with a Zeiss electron microscope EM912 (Carl Zeiss, Jena, Germany). Digital images were acquired with a Gatan Bioscan CCD camera (Gatan GmbH, München, Germany).

### Starch and chlorophyll quantification

Starch measurements were performed using an experimental protocol adapted from [41]. *C. reinhardtii *cells (about 2 × 10^6^) were harvested by centrifugation (13,000 *g*, 10 min). Cell pellets were resuspended in 1 mL methanol for chlorophyll extraction, mixed vigorously for 1 min and centrifuged. Supernatants were used to quantify chlorophyll photometrically [42], and pellets were left to dry in the hood to remove residual methanol. Pellets were then resuspended in 400 μL of distilled H_2_O, and autoclaved for 15 min at 120°C for starch solubilization. Total starch was quantified using an enzymatic starch assay kit including a commercial amyloglucosidase solution to convert starch to glucose (Sigma-Aldrich ref. SA-20, Saint Louis, USA). Glucose was quantified using an automated YSI 2700 select sugar analyzer (YSI Life Sciences, Yellow Springs, USA) using glucose as standard.

### Lipid analyses

Several methods of oil quantification are available in the literature including gravimetric measurement, glycerol analysis, NMR, or GC-FID. The most common is total lipid extraction with a Bligh and Dyer procedure [43], followed by separation on a Thin Layer Chromatography (TLC), recovery of the triacylglycerol (TAG) from the silica, transmethylation and quantification of the fatty acid methyl esters (FAMEs) by Gas Chromatography with Flame Ionization Detector (GC-FID). A drawback of this method is that it is tedious and time consuming. A simplified protocol using direct transmethylation of tissues [44] which is routinely used for biological material such as oilseeds (where TAGs represent the vast majority of lipids), might be applied to microalgal cultures but for screening purposes only. It is not suitable for precise quantification of TAGs in cells such as *C. reinhardtii *where oil might represent only a fraction of total cellular lipid content. A method for neutral lipid estimation in *C. reinhardtii *based on Nile red is available [45] but is mainly qualitative. The procedure we used is based on total lipid extraction, TLC and densitometry. It allows specific quantification of TAGs and can be used in medium throughput screenings.

#### Extraction of total cellular lipids

Lipids were extracted from *C. reinhardtii *cells by modified Bligh and Dyer [43]. Briefly, cells (usually 5 million) were harvested by centrifugation at 10,000 g for 10 min and 1 mL of 1 mM EDTA in 0.15 M acetic acid was added. The mixture was transferred to a glass tube with Teflon-lined screw cap and, after addition of 3 ml methanol:chloroform (2:1, v/v), was vortexed for 10 min with a multi-tube vortexer (VX-2500, VWR). Then, 1 mL of chloroform and 0.8 ml of KCl 0.88% (w/v) were added before vortexing and centrifuging at 4000 rpm for 2 min. The lower chloroform phase was transferred to a new glass tube. Cells were then extracted again with hexane, centrifuged and the supernatant was combined with the previous chloroform extracts. Lipid extracts were dried under a stream of N_2 _and re-suspended in solvent for HPTLC or GC-FID analysis.

#### Quantification of oil content by high performance-thin layer chromatography (HPTLC)

Triacylglycerols (TAGs) were quantified using a densitometry method by comparing to a standard curve generated from known amounts of triheptadecanoin (C17:0 TAG) standard. Typically, around 0.5 μg of lipid extract was loaded as a spot onto 20 × 20 cm silica gel 60 F254 HPTLC plates (Merck KGaA, Germany) using an ATS 5 automatic TLC sampler (Camag, Switzerland). Plates were then developed fully once in an ADC2 automatic developing chamber (Camag) using a hexane/diethyl ether/acetic acid (17/3/0.2, v/v/v) solvent mixture, thoroughly dried under the hood, dipped for 6s in a modified CuSO_4 _reagent [46] (20 g CuSO_4_, 200 ml methanol, 8 ml H_2_SO_4_, 8 ml H_3_PO_4_), heated at 141°C for 30 min on a TLC plate heater and finally scanned using a TLC Scanner 3 with WinCATs software (Camag). Results obtained by this densitometry procedure were not significantly different from those obtained by recovery of the TAG fraction from the TLC plate, transmethylation and quantification of fatty acid methyl esters by gas chromatography with flame ionization detector (see below). All polar lipids such as galactolipids were also quantified by HPTLC/densitometry. Plates were developed with an acetone/toluene/water (91/30/8, v/v/v) solvent mixture. Lipid extracts were run alongside purified polar lipid standards (Sigma-Aldrich, Saint-Louis, USA; Larodan Fine Chemicals AB, Malmö, Sweden).

#### Preparation of fatty acid methyl esters (FAMEs), identification by gas chromatography coupled to mass spectrometry (GC-MS) and quantification by gas chromatography with flame ionization detector (GC-FID)

FAMEs were prepared from Chlamydomonas total lipid extract in a glass tube with a Teflon-lined screw cap using acid-catalyzed transmethylation as described in Li et al. [44]. Internal standard used was triheptadecanoin (C17:0 TAG). FAMEs separation and identification was carried out on a FOCUS gas chromatograph equipped with DSQ II quadrupole mass spectrometer using the electron impact ionization mode (Thermo Fisher Scientific, San Jose, USA). A polar TR-WAX column ensuring good separation of polyunsaturated fatty acid species was used (Thermo Fisher Scientific; length 30 m, diameter 0.25 mm, film thickness 0.25 μm). Hydrogen was the carrier gas. The injector and transfer line were maintained at 225°C and 250°C respectively. The source temperature was 200°C. Samples were injected in split mode (5:1 split ratio) at an oven temperature of 45°C. After 1.5 min, the oven temperature was raised to 150°C at 15°C min^-1 ^then to 240°C at 6°C min^-1 ^and held at 240°C for 3 min. FAMEs were identified by retention time, fragmentation pattern and, when necessary, comparison with purified FAME standards (Larodan Fine Chemicals AB, Malmö, Sweden). FAMEs were quantified with a GC-FID (Thermo Fisher Scientific, San Jose, USA). Samples were injected in split mode (split ratio 1:5). Detector was set at 250°C. Carrier gas, column and oven temperature program were the same as for GC-MS analysis. For the TAG fraction, the fatty acid composition was determined as follows. TAGs were separated from other lipids by HPTLC as described above and the TAG fraction was revealed by spraying with primuline (0.005% in 80% acetone) and visualized under UV light. The silica corresponding to the TAG fraction was carefully scrapped off the TLC plate and packed into a Pasteur pipette plugged with a bit of glass wool. TAGs were eluted with a chloroform/methanol/H_2_O (5/5/1, v/v/v) mixture and a final rinse with hexane. The TAG extract was recovered, dried under a stream of N_2 _and FAMEs were prepared and analyzed by GC-MS or GC-FID.

## Authors' contributions

MS, FB, CT, YLB, GP designed the experiments. MS, SC, YLB, MN, BF, CC, AB, PC performed the experiments. YLB, FB, GP wrote the manuscript. All authors read and approved the manuscript.

## Supplementary Material

Additional file 1 Kinetics of oil accumulation in *Chlamydomonas reinhardtii *strain CC124 and *cw15 *(mean ± SD, n = 3) Click here for file

Additional file 2 **Changes in fatty acid composition in *C. reinhardtii *during N depletion (mean ± SD, n = 3)**. Click here for file

Additional file 3 **Mobilization of triacylglycerols and starch in strain *330 *after re-supply with nitrogen expressed on a per mL culture basis**. Cells were first cultivated in TAP medium until mid log phase, then transferred to TAP-N for 3 days under constant light and then switched to MM media in the dark. Values are mean of three independent experiments ± SD. Click here for file
