# Supplementary figures and images for: Oil accumulation in the model green alga Chlamydomonas reinhardtii: characterization, variability between common laboratory strains and relationship with starch reserves
Source: BMC Biotechnol. 2011 Jan 21;11:7. doi: 10.1186/1472-6750-11-7 (PMC3036615; doi:10.1186/1472-6750-11-7)

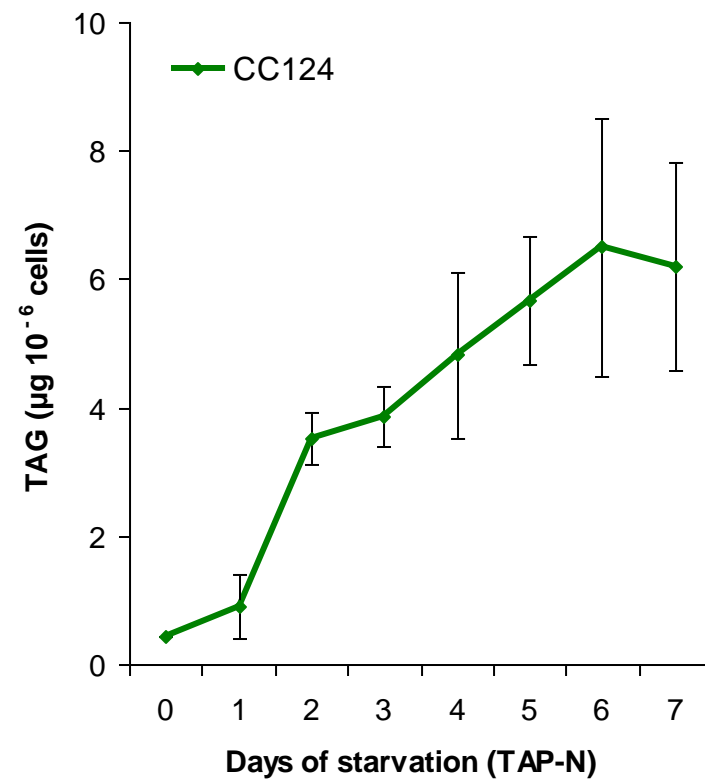

Supplement: Additional file 1 — Kinetics of oil accumulation in Chlamydomonas reinhardtii strain CC124 and cw15 (mean ± SD, n = 3) [file 1472-6750-11-7-S1.PDF]

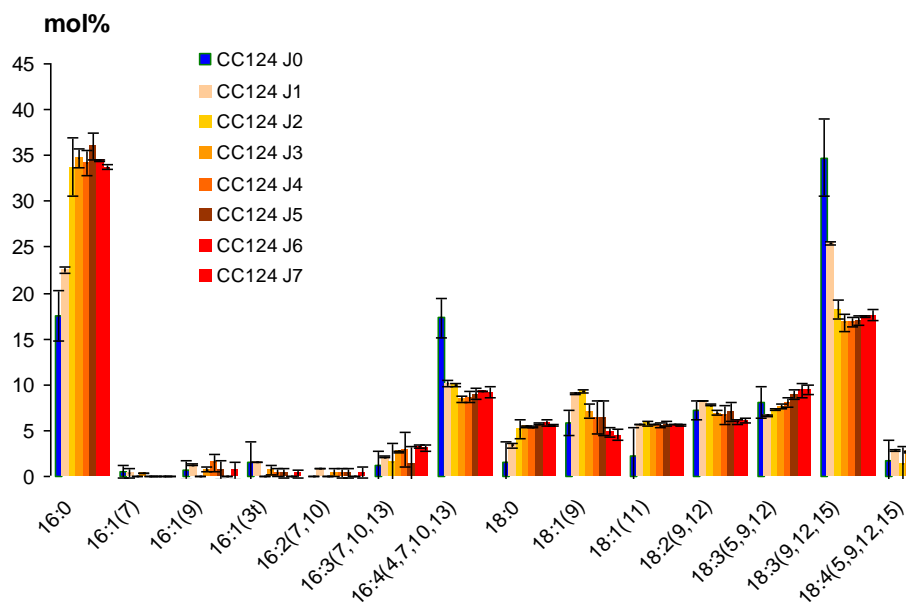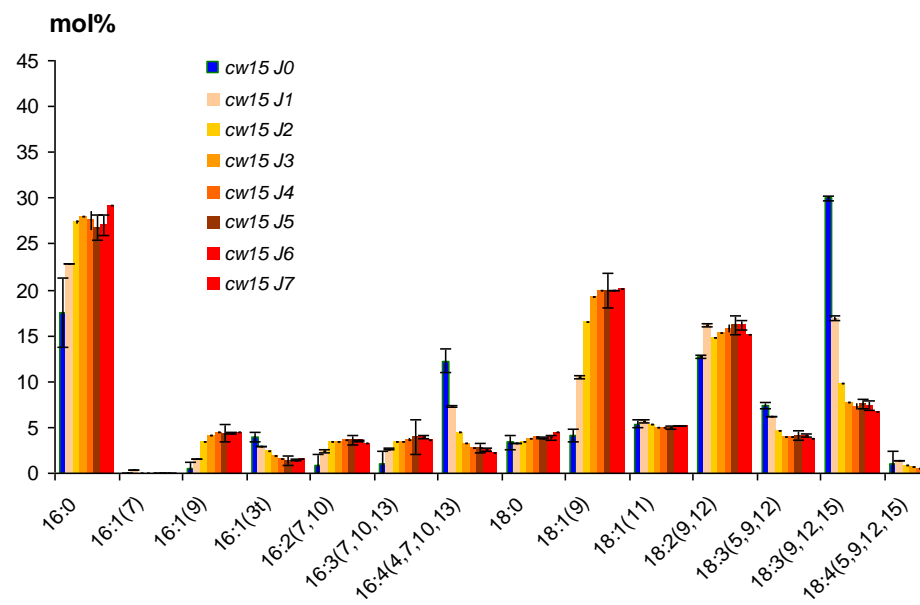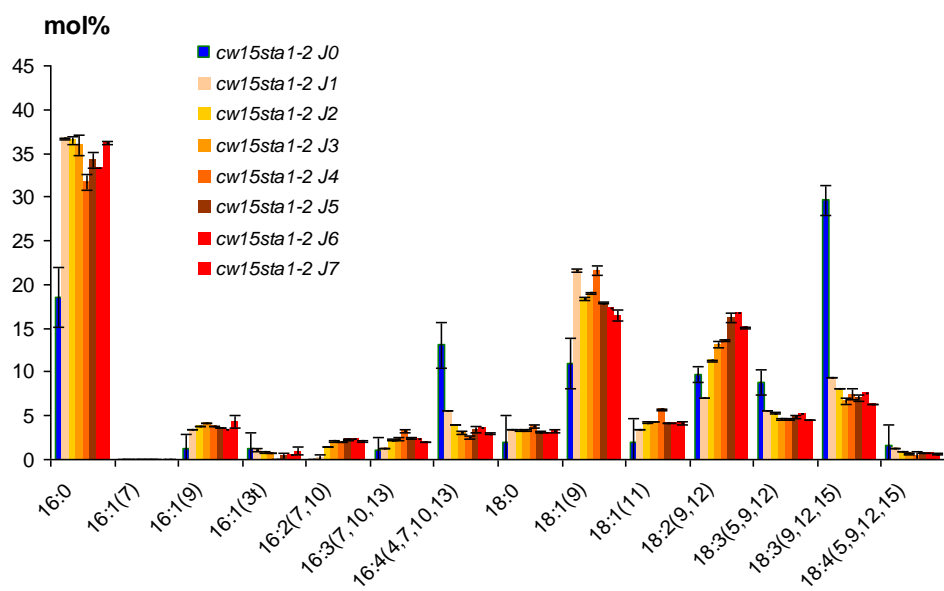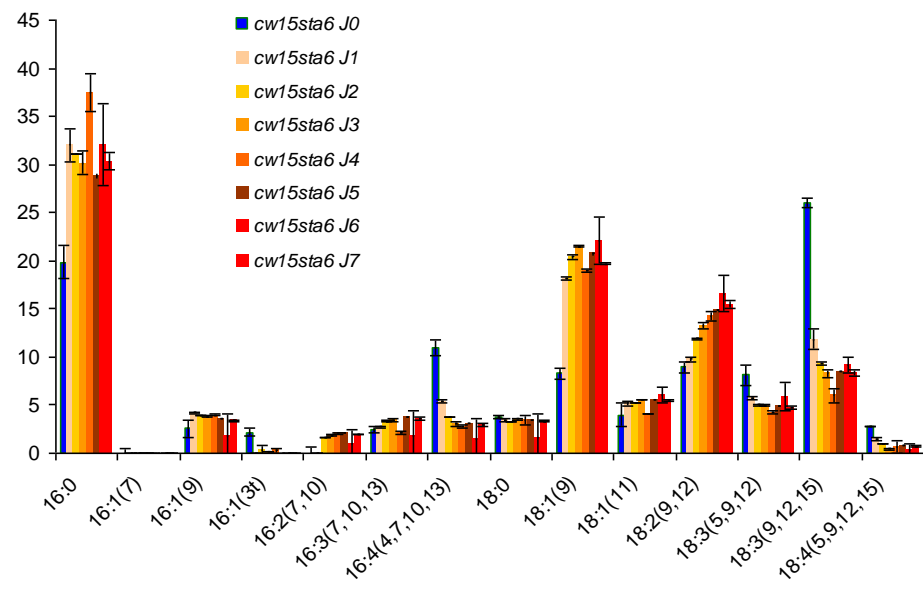

Supplement: Additional file 2 — Changes in fatty acid composition in C. reinhardtii during N depletion (mean ± SD, n = 3). [file 1472-6750-11-7-S2.PDF]

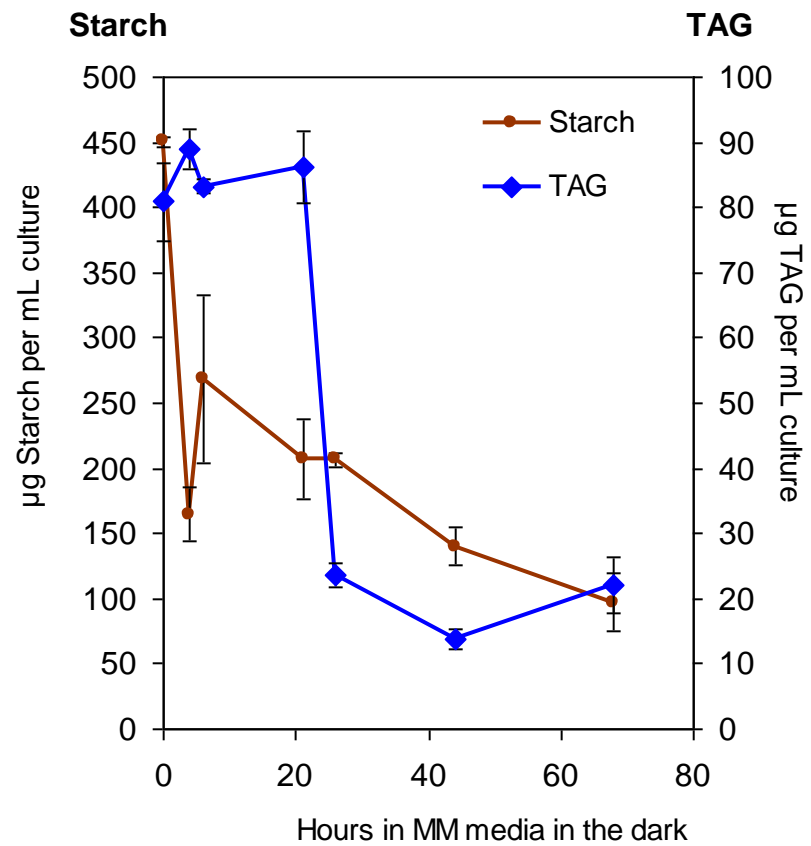

Supplement: Additional file 3 — Mobilization of triacylglycerols and starch in strain 330 after re-supply with nitrogen expressed on a per mL culture basis. Cells were first cultivated in TAP medium until mid log phase, then transferred to TAP-N for 3 days under constant light and then switched to MM media in the dark. Values are mean of three independent experiments ± SD. [file 1472-6750-11-7-S3.PDF]
